# The Association Between Self-Regulation and Daily Sports Activities in a Nationally Representative Sample of Older Adults. Findings From the German Ageing Survey

**DOI:** 10.3389/fphys.2018.01763

**Published:** 2018-12-04

**Authors:** André Hajek, Hans-Helmut König

**Affiliations:** Department of Health Economics and Health Services Research, Hamburg Center for Health Economics, University Medical Center Hamburg-Eppendorf, Hamburg, Germany

**Keywords:** self-control, sports, aged, middle aged, cohort study, cross-sectional, physical activity

## Abstract

There is a large body of literature about the determinants of exercising regularly in older adults. However, to date, there is limited evidence showing that self-regulation is associated with exercising regularly. Existing studies are mostly restricted to rather specific or small samples. Thus, the purpose of this study was to examine whether self-regulation is associated with daily sports activities among older adults. For the current study, cross-sectional data were used from the German Ageing Survey (*n* = 7,757), a nationally representative sample of community-dwelling individuals aged 40 and over in Germany. Logistic regression analysis showed that daily sports activities is positively associated with self-regulation [OR: 1.32 (95%-CI: 1.11–1.58)]. The present study highlights the importance of an association between daily sports activities and self-regulation. Knowledge about this association is useful in addressing cases where older adults exercise less than daily.

## Introduction

The number of older adults is expected to rise due to rapid demographic aging. In the second half of life, individuals experience loss of a spouse, loss of functional abilities, cognitive decline, and frailty or admission to a nursing home. Physical activities are often considered a protective factor against a range of negative health outcomes including morbidity and mortality (Paffenbarger et al., 1993; Fogelholm, 2010; Fontana, 2018; Nowak et al., 2018), meanwhile physical inactivity has been labeled a pandemic due to its consequences in various fields (e.g., health, economic, or social consequences) (Eijsvogels and Thompson, 2015). According to the “European Guidelines on cardiovascular disease prevention in clinical practice" (Perk et al., 2012) 30 min of moderate physical activity on most days per week are recommended. Consequently, exercising daily is of great importance. Older adults tend to exercise less than younger age groups (Macera et al., 2005). However, it should be acknowledged that other recommendations exist. Please see the recommendations provided by the WHO for further details (World Health Organization, 2010).

There is a large body of literature about the determinants of exercising regularly in older adults (Trost et al., 2002; Hajek et al., 2016). For example, it has been shown that regular exercise is positively associated with male gender, education, income, or activity history during adulthood (Trost et al., 2002). There is limited evidence showing that self-regulation is associated with exercising regularly (Umstattd and Hallam, 2007).

The concept of self-regulation can be described as an aspect of controlled processing and is based on the theory of selection, optimization, and compensation (SOC). This theory states that the three processes of SOC advance the maximization of gains and the minimization of losses related to aging. Goals important to the individual are followed through four self-management strategies (Ziegelmann and Lippke, 2007), namely: (i) elective selection (e.g., aim to develop an active lifestyle), (ii) optimization (e.g., conducting goal-directed actions), (iii) loss-based selection (e.g., changing the goal in reaction to losses), and (iv) compensation (use of means in reaction to losses). Further details related to this theory can be found elsewhere (Baltes and Carstensen, 1996).

In other words, individuals scoring low in self-regulation are not willing to postpone short-term needs (e.g., watching TV) to achieve long-term goals (stay healthy due to physical activity). To date, some studies have investigated the association between self-regulation and exercising regularly among older adults, showing that self-regulation is positively associated with regular exercise (Umstattd and Hallam, 2007; Son et al., 2009; Kosteli et al., 2018). For example, in the study conducted by Umstattd and Hallam (2007), regular exercise was defined as 3 or more days per week of moderate to vigorous exercise ( ≥ 30 min per day). However, these studies are mostly restricted to rather specific or small samples (please see the discussion section for further details regarding the samples) (Umstattd and Hallam, 2007; Son et al., 2009; Kosteli et al., 2018). Thus, the aim of the current study was to examine whether self-regulation is associated with daily sports activities using a nationally representative sample of community-dwelling individuals in the second half of life ( ≥ 40 years) in Germany.

## Materials and Methods

### Sample

For the present analysis, the data were derived from the fifth wave (2014) of the German Ageing Survey (DEAS). The first wave was carried out in 1996. Further waves took place in 2002 (second wave), 2008 (third wave), 2011 (fourth wave), and 2014. It is a nationally representative study of individuals in the second half of life (≥40 years) living in private households in Germany.

In sum, 7,757 individuals completed the drop-off questionnaire in 2014 and provided data on the frequency of sports activities as well as self-regulation. We restricted our analysis to the fifth wave because self-regulation was only quantified in this wave. Further details about the German Ageing Survey has been reported elsewhere (Klaus et al., 2017).

Prior to the interview, written informed consent was given. The German Ageing Survey complies with the Federal Data Protection Act. The principles outlined in the Declaration of Helsinki were followed. It is worth noting that an ethical statement for this study was not necessary as the criteria for requiring an ethical statement were not met (risk for the respondents, lack of information about the aims of the study, and examination of patients). Thus, ethics approval was not required as per the Institution’s guidelines and national regulations.

### Dependent Variable

In the questionnaire, it was asked “How often do you do sports such as hiking, soccer, gymnastics, or swimming?” (daily; several times a week; once a week; 1–3 times a month; less often; never). It is worth noting that the frequency format is based on the International Physical Activity Questionnaire (IPAQ) (Craig et al., 2003). The item used in the current study has been examined elsewhere (Wurm et al., 2010).

Because the aim of this study was to examine the association between self-regulation and daily sports activities among older adults, this variable was dichotomized (0 = several times a week; once a week; 1–3 times a month; less often; never; 1 = daily).

### Independent Variables

A validated scale by Ziegelmann and Lippke (2006) which was based on a scale originally developed by Freund and Baltes (2002) was used to quantify self-regulation, covering four items (1 = strongly agree; 2 = agree; 3 = disagree; 4 = strongly disagree). These items were: “I do everything I can to realize my plans,” “I have set my goals clearly and stick to them,” “When it becomes harder for me to get the same results, I keep trying harder until I can do it as well as before,” “When I can’t do something important the way I did before, I look for a new goal.” All items were reverse coded (i.e., 1 to 4; 2 to 3; 3 to 2; 4 to 1).

The scale is based on theory of SOC. The index score ranges from 1 to 4, with higher values corresponding to higher self-regulation. This 4-item scale (with 4 answer options) was also used in (in slightly adapted versions) in two previous studies (Reuter et al., 2010; Wurm et al., 2013). In these previous studies, Cronbach’s alpha was high (0.84 and 0.85, respectively). Cronbach’s alpha was 0.78 in our study.

Sex, age, and marital status [married and living together with spouse; others (widowed, single, divorced)], monthly net equivalent income in Euro, occupational status (working; retired; other: not employed), and region (West- vs. East Germany) were identified as potential confounders (socioeconomic covariates). Self-reported body-mass-index (weight in kilograms divided by squared height in meters), smoking behavior (yes, daily; yes, sometimes; no, not anymore; never been a smoker), and frequency of alcohol consumption (daily; several times a week; once a week; one to three times a month; less frequently; never) were also identified (behavioral covariates). Moreover, physical functioning (subscale ‘physical functioning’ of the SF-36, (Ware and Sherbourne, 1992) ranging from 0 = worst score to 100 = best score), self-rated health (from 1 = “very bad” to 5 = “very good”), depressive symptoms (Center for Epidemiological Studies Depression Scale, CES-D (Radloff, 1977); 15 items, ranging from 0 = no depressive symptoms to 15 = severe depressive symptoms), and the number of chronic illnesses (e.g., cardiac and circulatory disorders; stomach and intestinal problems; diabetes; eye problems, vision impairment; ear problems, hearing problems; ranging from 0 to 11) were identified as potential confounders (health covariates). The number of chronic illnesses was used to reflect the somatic burden.

### Statistical Analysis

First, sample characteristics stratified by daily sports activities (dichotomous) were examined (using *t*-tests and chi square tests, as appropriate). Second, multiple logistic regression analysis was used to investigate whether self-regulation is associated with daily sports activities, adjusting for potential confounders.

Third, as there is evidence that exercising at least several times a week is associated with positive health outcomes (Paffenbarger et al., 1986; Lindwall et al., 2007), we changed the focus from daily sports activities to frequent sports activities (0 = once a week; 1–3 times a month; less often; never; 1 = daily; several times a week) in sensitivity analysis. Fourth, regression analysis stratified by age (younger than 65 years; 65 years and over) were conducted. The level of significance was set at α = 0.05.

## Results

### Sample Characteristics

In the total sample, 50.9% of the individuals were female, and average age was 64.4 years (±11.2 years, 40–95 years). Sample characteristics stratified by daily sports activities (dichotomous) are depicted in Table 1. The relative frequency of each cell within its row was displayed for categorical and ordinal variables.

**Table 1:** Sample characteristics stratified by daily sports activities (*n* = 7,757).

	Frequency of sports activities		
	Individuals exercising daily (*n* = 646, 8.3%)	Individuals not exercising daily (*n* = 7,111, 91.7%)	
Independent variables	*N* (%)/Mean (±*SD*)	*N* (%)/Mean (±*SD*)	*p*-value
Sex: Male	301 (7.9)	3,508 (92.1)	0.18
Female	345 (8.7)	3,603 (91.3)	
Age in years	67.2 (±10.7)	64.2 (±11.2)	<0.001
Marital status: Single, divorced, widowed	209 (9.1)	2,098 (90.9)	0.13
Married, living together with spouse	436 (8.0)	4,998 (92.0)	
Monthly net equivalent income (€)	2,059.0 (±1,354.8)	1,935.7 (±1,388.8)	<0.05
Monthly net equivalent income (€) in categories: 0 € to <1,000 €	66 (7.4)	823 (92.6)	0.17
1,000 € to <2,000 €	286 (7.8)	3,398 (92.2)	
2,000 € to <3,000 €	164 (9.2)	1,628 (90.8)	
≥ 3,000 €	89 (9.2)	878 (90.8)	
Employment status: Employed	173 (6.1)	2,670 (93.9)	<0.001
Retired	427 (10.1)	3,786 (89.9)	
Other	46 (6.6)	651 (93.4)	
Region: East Germany	203 (8.0)	2,351 (92.0)	0.40
West Germany	443 (8.5)	4,760 (91.5)	
Body-Mass-Index	25.6 (±4.1)	27.0 (±4.6)	<0.001
Smoking behavior: Yes, daily	40 (3.8)	1,023 (96.2)	<0.001
Yes, sometimes	19 (6.2)	287 (93.8)	
No, not anymore	271 (9.5)	2,577 (90.5)	
Never been a smoker	309 (8.9)	3,168 (91.1)	
Frequency of alcohol consumption: Daily	71 (7.7)	851 (92.3)	0.76
Several times a week	146 (7.8)	1,728 (92.2)	
Once a week	100 (8.1)	1,130 (91.9)	
One to three times a month	78 (8.4)	852 (91.6)	
Less frequently	167 (9.0)	1,682 (91.0)	
Never	75 (8.7)	787 (91.3)	
Physical functioning (from 0 = worst score to 100 = best score)	82.6 (±23.0)	81.6 (±22.9)	0.32
Depressive symptoms (from 0 = no depressive symptoms to 15 = severe depressive symptoms)	5.9 (±5.6); median: 4; interquartile range: 6	6.7 (±6.0); median: 5; interquartile range: 7	<0.001
Self-rated health (from 1 = ,,very bad“ to 5 = ,,very good“)	3.6 (±0.9)	3.5 (±0.8)	<0.001
Number of chronic illnesses (from 0 to 11)	2.6 (±1.9)	2.6 (±1.9)	0.99
Self-regulation (from 1 to 4, with higher values correspond to higher self-regulation)	3.1 (±0.5)	3.0 (±0.5)	<0.001


*Comparisons between the two groups (individuals exercising daily; individuals not exercising daily) were done using *t*-test and chi-square procedures. SD, standard deviation.*

### Multiple Regression Analysis

The results of multiple logistic regression analysis (main analysis) are displayed in Table 2.

**Table 2:** Determinants of daily sports activities (0 = several times a week; once a week; 1–3 times a month; less often; never; 1 = daily).

Independent variables	Daily sports activities
Sex (Ref.: Male)	0.99
	(0.82–1.21)
Age in years	1.00
	(0.99–1.02)
Other marital status (Ref.: Married, living together with spouse)	1.17
	(0.96–1.42)
Monthly net equivalent income (€)	1.00+
	(1.00–1.00)
Employment status: Retired (Ref.: Employed)	1.62^∗∗^
	(1.18–2.23)
Other (not employed)	1.21
	(0.82–1.78)
Region: East Germany (Ref.: West Germany)	0.98
	(0.81–1.18)
Body-Mass-Index	0.92^∗∗∗^
	(0.90–0.94)
Smoking behavior: Yes, sometimes (Ref.: Yes, daily)	1.96^∗^
	(1.08–3.56)
No, not anymore	2.64^∗∗∗^
	(1.80–3.87)
Never been a smoker	2.29^∗∗∗^
	(1.57–3.34)
Frequency of alcohol consumption: Several times a week (Ref.: Daily)	1.23
	(0.88–1.70)
Once a week	1.36+
	(0.95–1.93)
One to three times a month	1.35
	(0.92–1.97)
Less frequently	1.52^∗^
	(1.09–2.12)
Never	1.65^∗^
	(1.13–2.42)
Physical functioning (from 0 = worst score to 100 = best score)	1.00
	(0.99–1.00)
Depressive symptoms (from 0 = no depressive symptoms to 15 = severe depressive symptoms)	0.99
	(0.97–1.01)
Self-rated health (from 1 = ,,very bad“ to 5 = ,,very good“)	1.20^∗^
	(1.04–1.38)
Number of chronic illnesses (from 0 to 11)	1.04
	(0.98–1.10)
Self-regulation (from 1 to 4, with higher values corresponding to higher self-regulation)	1.32^∗∗^
	(1.11–1.58)
Constant	0.03^∗∗∗^
	(0.01–0.14)
Observations	6,841
Pseudo *R*^2^	0.04


*Results of multiple logistic regression analysis. Odds ratios (95% confidence intervals) are displayed. ^∗∗∗^*p* < 0.001, ^∗∗^*p* < 0.01, ^∗^*p* < 0.05, +*p* < 0.10.*

Logistic regression analysis showed that daily sports activities were positively associated with self-regulation [OR: 1.32 (95%-CI: 1.11–1.58)]. Please refer to Table 2 regarding potential confounders.

As it has been demonstrated that exercising at least several times a week is associated with positive health outcomes, (Paffenbarger et al., 1986; Lindwall et al., 2007) we changed the focus from daily sports activities to frequent sports activities (0 = once a week, 1–3 times a month, less often, never; 1 = daily, several times a week) in an additional analysis (Table 3). The findings remained similar. For example, the positive association between at least exercising several times a week and self-regulation remained significant [OR: 1.18 (1.06–1.31)].

**Table 3:** Determinants of frequent sports activities (0 = once a week; 1–3 times a month; less often; 1 = several times a week or daily).

Independent variables	Frequent sports activities
Sex (Ref.: Male)	1.20^∗∗^
	(1.07–1.35)
Age in years	0.98^∗∗∗^
	(0.97–0.98)
Other marital status (Ref.: Married, living together with spouse)	1.04
	(0.93–1.17)
Monthly net equivalent income (€)	1.00^∗∗∗^
	(1.00–1.00)
Employment status: Retired (Ref.: Employed)	1.80^∗∗∗^
	(1.49–2.16)
Other (not employed)	1.35^∗∗^
	(1.09–1.67)
Region: East Germany (Ref.: West Germany)	0.66^∗∗∗^
	(0.58–0.74)
Body-Mass-Index	0.94^∗∗∗^
	(0.93–0.95)
Smoking behavior: Yes, sometimes (Ref.: Yes, daily)	1.86^∗∗∗^
	(1.36–2.56)
No, not anymore	2.72^∗∗∗^
	(2.24–3.30)
Never been a smoker	2.52^∗∗∗^
	(2.09–3.05)
Frequency of alcohol consumption: Several times a week (Ref.: Daily)	1.27^∗^
	(1.06–1.52)
Once a week	1.47^∗∗∗^
	(1.20–1.79)
One to three times a month	1.25^∗^
	(1.01–1.55)
Less frequently	1.08
	(0.89–1.32)
Never	1.06
	(0.84–1.34)
Physical functioning (from 0 = worst score to 100 = best score)	1.01^∗∗∗^
	(1.00–1.01)
Depressive symptoms (from 0 = no depressive symptoms to 15 = severe depressive symptoms)	0.99^∗^
	(0.98–1.00)
Self-rated health (from 1 = ,,very bad“ to 5 = ,,very good“)	1.21^∗∗∗^
	(1.11–1.31)
Number of chronic illnesses (from 0 to 11)	1.02
	(0.99–1.06)
Self-regulation (from 1 to 4, with higher values corresponding to higher self-regulation)	1.18^∗∗^
	(1.06–1.31)
Constant	0.35^∗^
	(0.14–0.84)
Observations	6,841
Pseudo *R*^2^	0.08


*Results of multiple logistic regression analysis. Odds ratios (95% confidence intervals) are displayed. ^∗∗∗^*p* < 0.001, ^∗∗^*p* < 0.01, ^∗^*p* < 0.05, ^+^*p* < 0.10.*

In addition, regression analysis stratified by age (<65 years; ≥65 years) were conducted. Among individuals aged 40–64 years, daily sports activities were positively associated with self-regulation [OR: 1.53 (1.15–2.05)]. Among individuals aged 65 years and over, this association was marginally significant [OR: 1.21 (0.97–1.51)].

## Discussion

Based on a nationally representative sample of individuals in the second half of life living in a private household, the purpose of the current cross-sectional study was to examine whether self-regulation is associated with daily sports activities. Logistic regression analysis showed that daily sports activities is positively associated with self-regulation.

A previous study demonstrated that various factors (e.g., marital status, gender, and education) were associated with regular exercise among a small convenience sample (*n* = 98) of older adults (Umstattd and Hallam, 2007) bivariately. After adjusting for potential confounders, only self-regulation was independently associated with regular exercise. Self-regulation was assessed with a 43-items questionnaire created by Petosa (1993).

Using a convenience sample (*n* = 271) among individuals aged 50 and over, a further cross-sectional study (Son et al., 2009) yielded similar results (global self-regulation was measured using the short version SOC Questionnaire) (Baltes et al., 1995). In addition, a recently published study (Kosteli et al., 2018) has also shown that self-regulation and physical activity are positively correlated with each other (bivariately) among older adults (*n* = 299, aged 50 and over; recruited via word of mouth, social media and posters). In the study conducted by Kosteli et al. (2018), self-regulation was quantified using the Exercise Planning and Scheduling Scale and the Exercise Goal-Setting Scale (Rovniak et al., 2002).

As this study used convenience-sampling methods, the sample is unlikely to be representative of the population being studied. There is a lack of knowledge regarding self-regulation and the frequency of sports activities based on nationally representative samples. Thus, extending previous knowledge based on relatively small and specific samples, this study demonstrated that self-regulation and daily sports activities are associated among older individuals living in private households in Germany.

Consequently, one strength of the present study is that data were gathered from a nationally representative study of community-dwelling older adults (≥40 years). Another strength is that self-regulation was quantified using a scale with established psychometric properties. However, we cannot dismiss the possibility that a sample selection bias is present in this study, although it has been shown that this bias is small (Klaus et al., 2017). Caution should be used in generalizing our findings to individuals residing in institutional settings. Self-reported frequency of sports activities was used, which has acknowledged limitations. In the DEAS study, we cannot distinguish between different sports actions. A correlational study was undertaken due to the nature of the data available, which could be considered a further limitation. Longitudinal studies are needed to analyze the relation between self-regulation and frequency of sports activities. Furthermore, it is worth noting that outcome expectancies were not assessed (Williams et al., 2005).

## Conclusion

The present study highlights the importance of an association between daily sports activities and self-regulation. Knowledge about this association is useful in addressing cases where older adults exercise less than daily. This is important as lesser physical activity can lead to functional and cognitive decline in the long term (Landi et al., 2007; Sofi et al., 2011).

As older adults usually have regular general practitioner (GP) visits, GPs might play a key role in identifying individuals at risk. For this purpose, screening for self-regulation among older patients in GP practices is important.

## Data Availability

The data used in this study are third-party data. The anonymized data sets of the DEAS (1996, 2002, 2008, 2011, and 2014) are available for secondary analysis. The data has been made available to scientists at universities and research institutes exclusively for scientific purposes. The use of data is subject to written data protection agreements. Microdata of the German Ageing Survey (DEAS) is available free of charge to scientific researchers for non-profitable purposes. The FDZ-DZA provides access and support to scholars interested in using DEAS for their research. However, for reasons of data protection, signing a data distribution contract is required before data can be obtained. Please see for further Information (data distribution contract): https://www.dza.de/en/fdz/access-to-data/formular-deas-en-english.html.

## Author Contributions

AH and H-HK designed and conceived the analyses, prepared the data, performed the statistical analysis and interpreted the data, and prepared the manuscript. Both authors critically reviewed the manuscript, provided significant editing of the article, and approved the final manuscript.

## Conflict of Interest Statement

The authors declare that the research was conducted in the absence of any commercial or financial relationships that could be construed as a potential conflict of interest.
